# Dopaminergic and muscarinic receptor involvement in neuromedin U-induced suppression of food intake in broiler chickens

**DOI:** 10.1016/j.psj.2026.108017

**Published:** 2026-10-22

**Authors:** Armin Ahmadi, Shahin Hassanpour, Morteza zendehdel, Mohaya Farzin

**Affiliations:** a Department of Veterinary Basic Sciences, SR.C., Islamic Azad University, Tehran, Iran; b Department of Basic Sciences, Faculty of Veterinary Medicine, University of Tehran, Tehran, Iran; c Razi Clinical Research Development Center, Guilan University of Medical Sciences, Rasht, Iran

**Keywords:** Neuromedin U, Broiler chickens, Appetite regulation, Dopaminergic receptors, Muscarinic receptors, Food intake

## Abstract

Neuromedin U (NMU) is a highly conserved neuropeptide involved in the regulation of appetite, energy homeostasis, and neuroendocrine functions. Although the anorexigenic effects of NMU have been demonstrated in both mammals and birds, the neurotransmitter pathways mediating these effects in poultry remain poorly understood. This study investigated the role of dopaminergic and muscarinic receptors in NMU-induced regulation of food intake in broiler chickens. Food-deprived broiler chicks received intracerebroventricular (ICV) injections of NMU and selective antagonists of dopamine (D1–D4) and muscarinic (M1–M4) receptors. Cumulative food intake was measured at 30, 60, and 120 min following treatment, and data were analyzed using repeated-measures two-way ANOVA followed by Duncan’s multiple range test. Central administration of NMU significantly reduced food intake in a dose-dependent manner. Pretreatment with the D1 receptor antagonist SCH23390 and the D2 receptor antagonist AMI-193 significantly attenuated NMU-induced hypophagia, whereas D3 and D4 receptor antagonists failed to modify this response. Similarly, antagonists of M1 (Telenzepine), M2 (AF-DX 116), and M3 (4-DAMP) receptors significantly reversed NMU-induced suppression of feeding, while M4 receptor blockade had no significant effect. These findings indicate that dopaminergic D1 and D2 and muscarinic M1, M2, and M3 receptor systems contribute to the hypophagic response induced by central NMU administration in broiler chicks, whereas blockade of D3, D4, and M4 receptors did not significantly alter this response. These findings provide evidence for receptor subtype-specific involvement in the central regulation of feeding by NMU in avian species and provide a basis for further investigation of the underlying neurochemical mechanisms.

## Introduction

Regulation of food intake is a complex physiological process controlled by the integration of central and peripheral signals that coordinate energy homeostasis, growth, and metabolic functions. In poultry, feed intake is one of the most important determinants of growth performance and production efficiency (Cao et al., 2020; Teranishi and Hanada, 2021). Therefore, understanding the neurobiological mechanisms that regulate appetite is of considerable importance for both basic research and the poultry industry.

Neuromedin U (NMU) is a highly conserved neuropeptide that was originally isolated from porcine spinal cord and subsequently identified in a wide range of vertebrate species (Greene et al., 2022; Ghashghayi et al., 2024). NMU is expressed in both the central nervous system and peripheral tissues, particularly within the gastrointestinal tract, and has been implicated in numerous physiological processes including smooth muscle contraction, stress responses, energy balance, and feeding regulation (Zhang et al., 2023; Saneyasu, 2024) .Previous studies have demonstrated that central administration of NMU produces a potent anorexigenic effect in mammals, leading to reduced food intake and body weight gain (Kaiya, 2024; Greene et al., 2020). Similar effects have also been reported in avian species, suggesting that NMU acts as an important regulator of feeding behavior across vertebrates (Ghashghayi et al., 2024; Mahdavi et al., 2024).

Accumulating evidence indicates that the anorexigenic action of NMU involves interactions with several neurotransmitter systems within the brain (Ghashghayi et al., 2024). Among these, dopaminergic signaling plays a critical role in the regulation of feeding motivation, reward processing, and energy balance (Saneyasu, 2024; Wallace and Fordahl, 2022). Dopamine receptors are widely distributed throughout hypothalamic and mesolimbic pathways and have been shown to influence both homeostatic and hedonic aspects of food intake (Baik, 2021; Onimus et al., 2026). Likewise, the cholinergic system, particularly muscarinic acetylcholine receptors, contributes significantly to appetite regulation and metabolic control (Tran et al., 2022; Deem et al., 2022). Different muscarinic receptor subtypes have been implicated in hypothalamic signaling pathways associated with feeding behavior and energy expenditure (Teranishi and Hanada, 2021; Saneyasu, 2024).

Although the appetite-suppressing effects of NMU have been documented in birds, the neurochemical mechanisms underlying these effects remain poorly understood (Ghashghayi et al., 2024; Mahdavi et al., 2024). In particular, little information is available regarding the contribution of dopaminergic and muscarinic receptor subtypes to NMU-induced regulation of food intake in broiler chickens. Elucidating these mechanisms may improve our understanding of central appetite-regulating pathways in avian species and provide valuable insights into the neuroendocrine control of feeding.

Therefore, the present study was designed to investigate the involvement of dopaminergic (D1–D4) and muscarinic (M1–M4) receptors in NMU-induced suppression of food intake in broiler chickens using intracerebroventricular administration of selective receptor antagonists. Although previous work has demonstrated that central dopaminergic, serotonergic, and GABAergic systems contribute to NMU-induced hypophagia in neonatal chicks, the receptor-subtype specificity of these responses has not been comprehensively characterized. In particular, the individual contributions of dopaminergic D1–D4 and muscarinic M1–M4 receptor subtypes have not been systematically evaluated in broiler chickens. Therefore, the present study was designed to characterize the receptor-subtype involvement using selective pharmacological antagonists. We hypothesized that specific dopaminergic and muscarinic receptor subtypes contribute to the anorexigenic effects of NMU and mediate its central regulation of feeding behavior.

## Materials and methods

### Animals and housing

One-day-old male broiler chicks (Ross 308) were obtained from a commercial hatchery and housed under continuous lighting conditions. Chicks were maintained in groups for the first two days and subsequently transferred to individual cages equipped with separate feeders. Feed and water were provided ad libitum throughout the experimental period, except during designated fasting periods before testing. Ambient temperature was maintained at 31 ± 2°C. A commercial starter diet containing 22% crude protein and 3000 kcal/kg metabolizable energy was provided throughout the study. All experimental procedures involving animals were conducted in accordance with the institutional guidelines for the care and use of laboratory animals and were approved by Research Committee of the Islamic Azad University, Science and Research Branch, Tehran, Iran (IR.IAU.SRB.REC.1403.255;).

### Drugs

Chicken neuromedin U (NMU) was used as the experimental peptide. The following receptor antagonists were employed: SCH23390 (dopamine D1 receptor antagonist), AMI-193 (dopamine D2 receptor antagonist), NGB2904 (dopamine D3 receptor antagonist), l-741,742 (dopamine D4 receptor antagonist), Telenzepine (muscarinic M1 receptor antagonist), AF-DX 116 (muscarinic M2 receptor antagonist), and 4-DAMP (muscarinic M3 receptor antagonist). PD102807 PD102807 (selective muscarinic M4 receptor antagonist, 125 nmol). All drugs were prepared immediately before administration according to the experimental protocol and were administered intracerebroventricularly in a total volume of 10 μL per chick. All compounds were dissolved or diluted in 0.85% physiological saline. Evans Blue (0.1%) was used to verify the accuracy of intracerebroventricular injections.

### Intracerebroventricular (ICV) injection procedure

Intracerebroventricular injections were performed in 5-day-old chicks according to the method described previously. Intracerebroventricular injections were performed in 5-day-old chicks according to a previously described ICV injection method (Jonaidi et al., 2012). Briefly, conscious chicks were restrained in a custom-made acrylic head holder positioned at a 45° angle. A Hamilton microsyringe (10 μL) was inserted through a predefined point on the skull to a depth of approximately 4 mm for delivery into the right lateral ventricle. The total injection volume was 10 μL per chick. Following completion of the experiments, chicks were euthanized and brains were removed for verification of injection placement. Only data obtained from birds showing Evans Blue dye localization within the lateral ventricle were included in the statistical analyses.

### Experimental design

Chicks were food-deprived for 3 h before each experiment. Following treatment administration, birds were returned to their cages with free access to feed and water. Cumulative food intake was measured at 30, 60, and 120 min post-injection and expressed as a percentage of body weight. All experiments be repeated at least twice. Experiments 1–8 was conducted as separate experimental trials using distinct cohorts of chicks. Each experiment included its own control and treatment groups, and food intake was measured independently at 30, 60, and 120 min after ICV administration. Animals included in one experiment were not reused in another experiment.

### Experiment 1: dose–response effect of NMU

Chicks were randomly assigned to four treatment groups: control (saline + 0.1% Evans Blue), NMU (0.1 μg), NMU (1 μg), and NMU (10 μg). The 10 μg dose was subsequently selected as the effective NMU dose for the receptor antagonist experiments. Each group included 11 chicks (n = 44 in experiment).

### Experiment 2: role of dopamine D1 receptors

The involvement of D1 receptors was examined using SCH23390 (5 nmol). Chicks were randomly assigned to four treatment groups: control, NMU, SCH23390, and NMU + SCH23390. The NMU group received the effective dose selected from Experiment 1. Each group included 11 chicks (n = 44 in experiment).

### Experiment 3: role of dopamine D2 receptors

The involvement of D2 receptors was examined using AMI-193 (5 nmol). Chicks were randomly assigned to four treatment groups: control, NMU, AMI-193, and NMU + AMI-193. Each group included 11 chicks (n = 44 in experiment).

### Experiment 4: role of dopamine D3 receptors

The involvement of D3 receptors was examined using NGB2904 (6.4 nmol). Chicks were assigned to four treatment groups: control, NMU (10 μg), NGB2904 (6.4 nmol), and NGB2904 + NMU. Each group included 11 chicks (n = 44 in experiment).

### Experiment 5: role of dopamine D4 receptors

The involvement of D4 receptors was examined using l-741,742 6 nmol). Chicks were randomly assigned to four treatment groups: control, NMU, l-741,742, and NMU + l-741,742. Each group included 11 chicks (n = 44 in experiment).

### Experiment 6: role of muscarinic M1 receptors

The involvement of M1 receptors was examined using Telenzepine (125 nmol). Chicks were assigned to four treatment groups: control, NMU (10 μg), Telenzepine (125 nmol), and Telenzepine + NMU. Each group included 11 chicks (n = 44 in experiment).

### Experiment 7: role of muscarinic M2 receptors

The involvement of M2 receptors was examined using AF-DX116 (125 nmol). Chicks were assigned to four treatment groups: control, NMU (10 μg), AF-DX116 (125 nmol), and AF-DX116 + NMU. Each group included 11 chicks (n = 44 in experiment).

### Experiment 8: role of muscarinic M4 receptors

The involvement of M3 receptors was examined using 4-DAMP (125 nmol). Chicks were assigned to four treatment groups: control, NMU (10 μg), 4-DAMP (125 nmol), and 4-DAMP + NMU. Each group included 11 chicks (n = 44 in experiment).

### Statistical analysis

The experiment was conducted using a completely randomized design. Food intake measured at 30, 60, and 120 min was analyzed using a two-way repeated-measures analysis of variance (ANOVA), with treatment as the between-subject factor and time as the within-subject repeated-measures factor. The treatment × time interaction was included in the model to determine whether the effect of treatment differed across time points. When appropriate, assumptions of the repeated-measures analysis, including sphericity, were evaluated and an appropriate correction was applied when the assumption was violated. Post-hoc comparisons were performed using Tukey’s test with an appropriate adjustment for multiple comparisons. Statistical significance was set at P < 0.05. Data are presented as mean ± SEM.

### Effect of neuromedin U on food intake

Intracerebroventricular administration of NMU significantly reduced cumulative food intake in neonatal broiler chicks in a dose-dependent manner. The highest dose of NMU (10 μg) produced the greatest suppression of feeding compared with the control group at 30-, 60-, and 120-min post-injection (P < 0.05). Food intake in chicks receiving 1 μg NMU was significantly lower than that of the control group but higher than that observed in the 10 μg NMU group. The lowest dose (0.1 μg) induced a moderate reduction in food consumption compared with the control treatment. Based on these findings, the 10 μg dose was selected as the effective dose for subsequent experiments (Fig. 1).

**Fig. 1:**
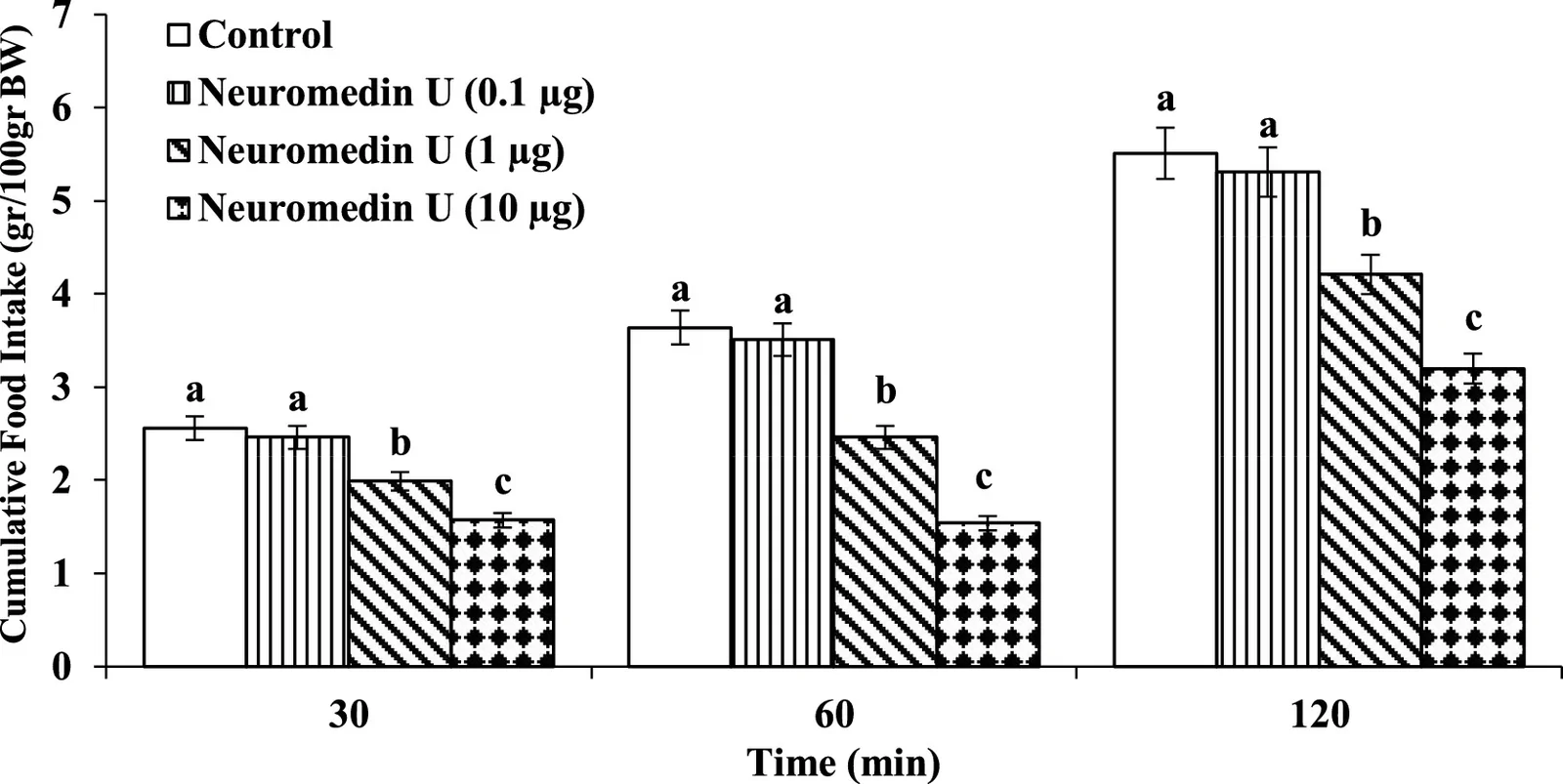
Effect of ICV injection of control solution and different levels of the Neuromedin U (0.1, 1, 10 µg) on cumulative food intake (gr/100gr BW) in neonatal broiler chicks (n = 44). Data are expressed as mean±SEM. Different letters (a, b and c) indicate significant differences between treatments at each time (P < 0.05).

### Involvement of dopamine D1 receptors

Administration of the D1 receptor antagonist SCH23390 alone did not significantly affect food intake compared with the control group. However, co-administration of SCH23390 with NMU significantly attenuated NMU-induced hypophagia at all measured time points (30, 60, and 120 min; P < 0.05). These findings suggest that D1 receptor signaling contributes to the hypophagic response induced by NMU. Nevertheless, food intake in the combined treatment group remained lower than that of the control group, indicating a partial reversal of the anorexigenic effect of NMU (Fig. 2).

**Fig. 2:**
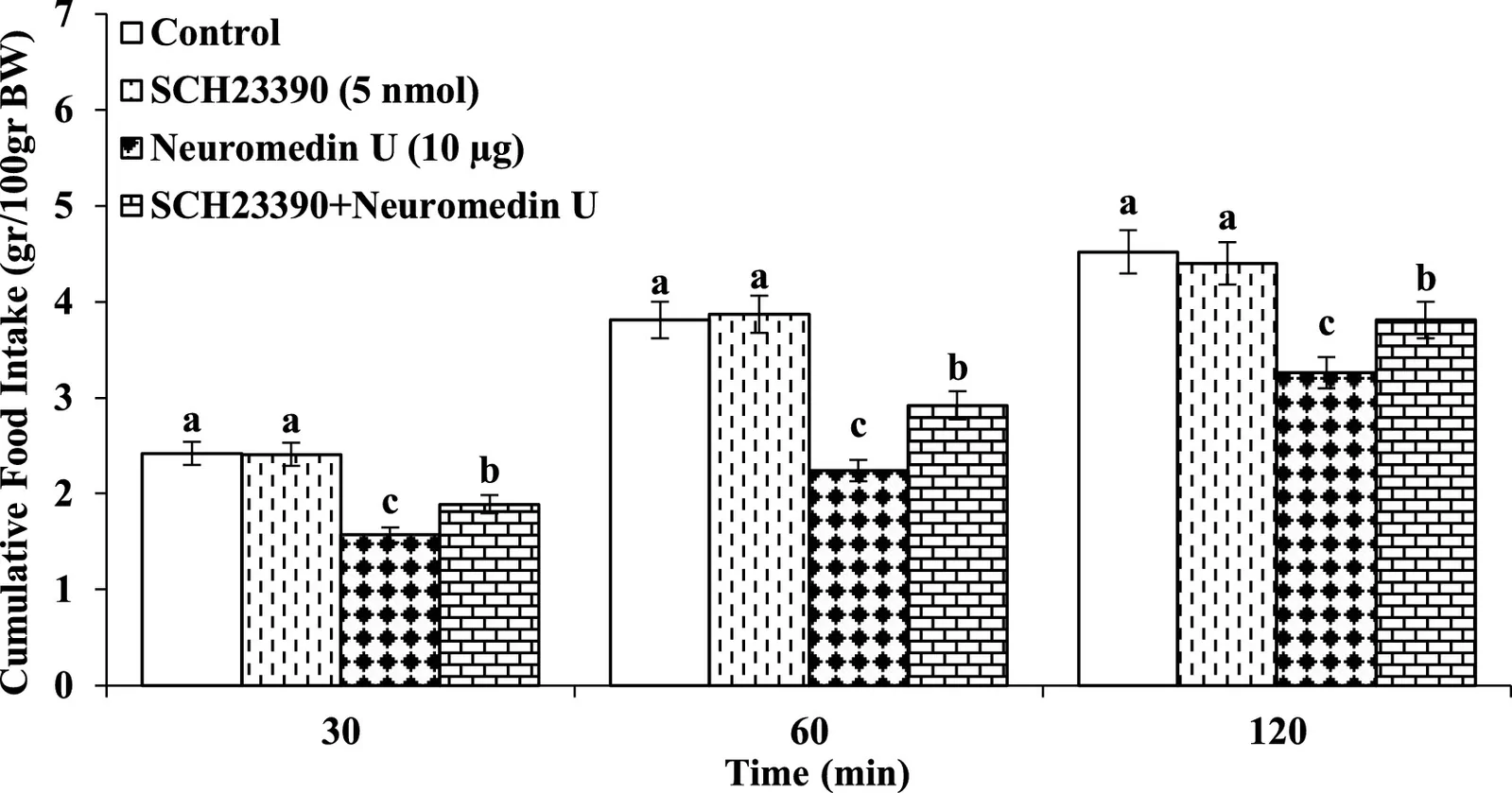
Effect of ICV injection of control solution, SCH23390 (D1 receptor antagonist, 5 nmol), Neuromedin U (10 µg) and a combination of SCH23390 plus Neuromedin U on cumulative food intake (gr/100gr BW) in neonatal broiler chicks (n = 44). Data are expressed as mean±SEM. Different letters (a, b and c) indicate significant differences between treatments at each time (P < 0.05).

### Involvement of dopamine D2 receptors

Similar to the D1 receptor antagonist, administration of AMI-193 alone did not alter cumulative food intake. Pretreatment with AMI-193 significantly reduced the suppressive effect of NMU on feeding behavior. Chicks receiving the combined AMI-193 + NMU treatment consumed significantly more feed than those receiving NMU alone (P < 0.05), Suggesting that D2 receptor signaling contributes to the hypophagic response induced by NMU (Fig. 3).

**Fig. 3:**
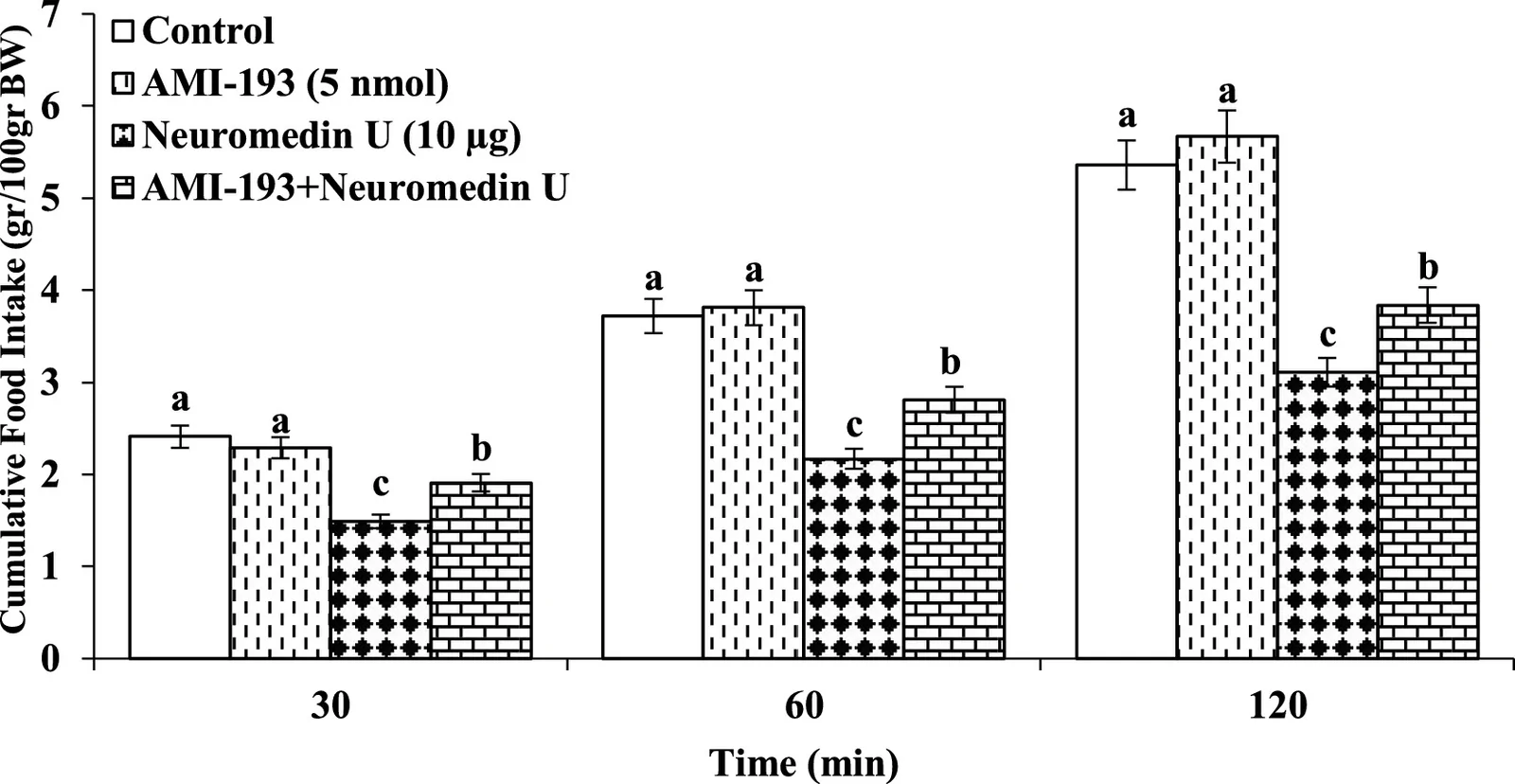
Effect of ICV injection of control solution, AMI-193 (D2 receptor antagonist, 5 nmol), Neuromedin U (10 µg) and a combination of AMI-193 plus Neuromedin U on cumulative food intake (gr/100gr BW) in neonatal broiler chicks (n = 44). Data are expressed as mean±SEM. Different letters (a and b) indicate significant differences between treatments at each time (P < 0.05).

### Involvement of dopamine D3 receptors

The D3 receptor antagonist NGB2904 did not significantly modify the anorexigenic action of NMU. Food intake in the NGB2904 + NMU group was not significantly different from that observed in the NMU-treated group at any recording interval. These findings indicate that D3 receptors are not critically involved in NMU-induced suppression of food intake in broiler chicks (Fig. 4).

**Fig. 4:**
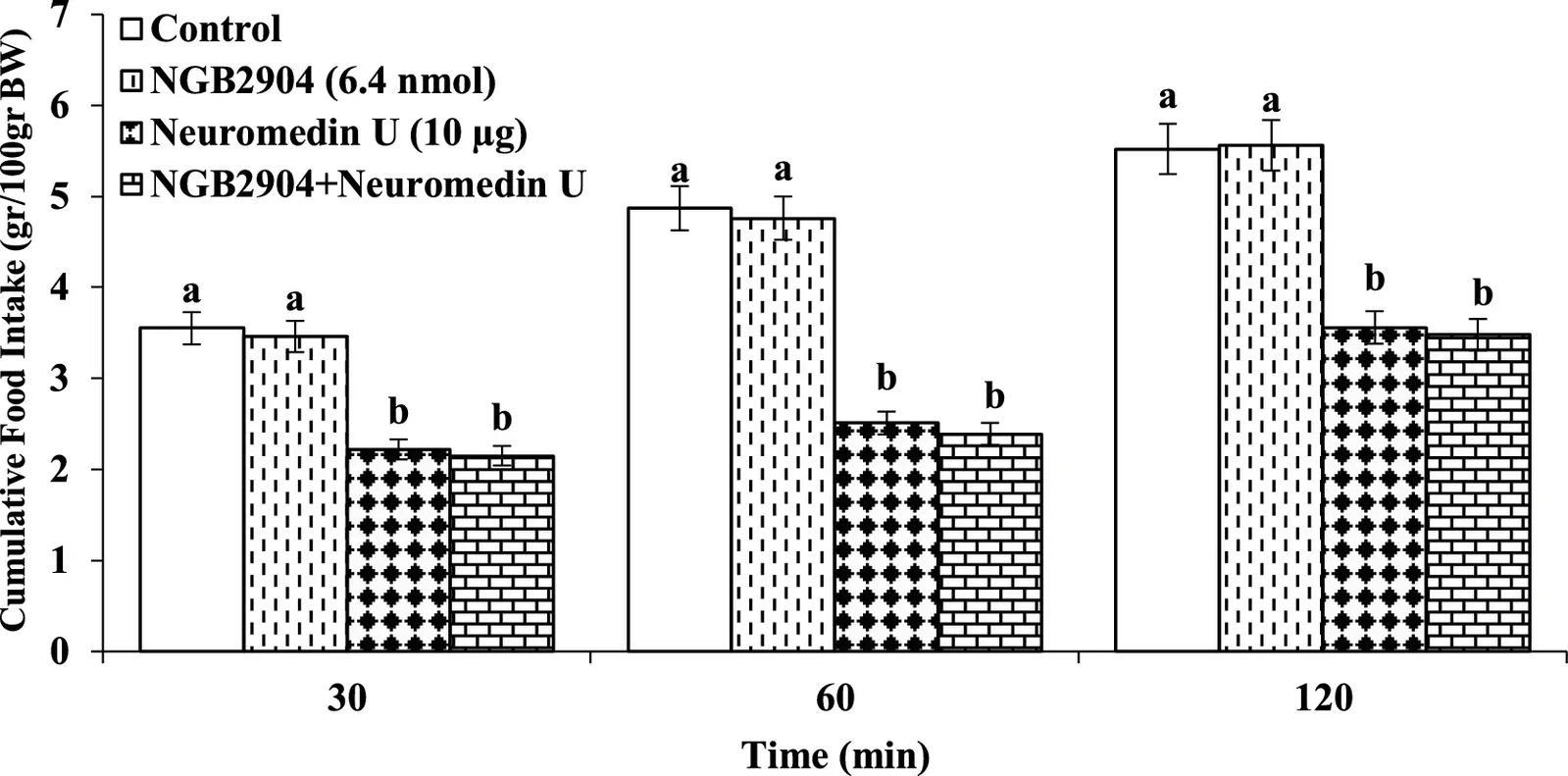
Effect of ICV injection of control solution, NGB2904 (D3 receptor antagonist, 5 nmol), Neuromedin U (10 µg) and a combination of NGB2904 plus Neuromedin U on cumulative food intake (gr/100gr BW) in neonatal broiler chicks (n = 44). Data are expressed as mean±SEM. Different letters (a and b) indicate significant differences between treatments at each time (P < 0.05).

### Involvement of dopamine D4 receptors

Likewise, administration of the D4 receptor antagonist l-741,742 failed to alter the hypophagic response induced by NMU. No significant differences were observed between the NMU group and the NMU + l-741,742 group, indicating that D4 receptor signaling does not play a major role in mediating the anorexigenic effects of NMU (Fig. 5).

**Fig. 5:**
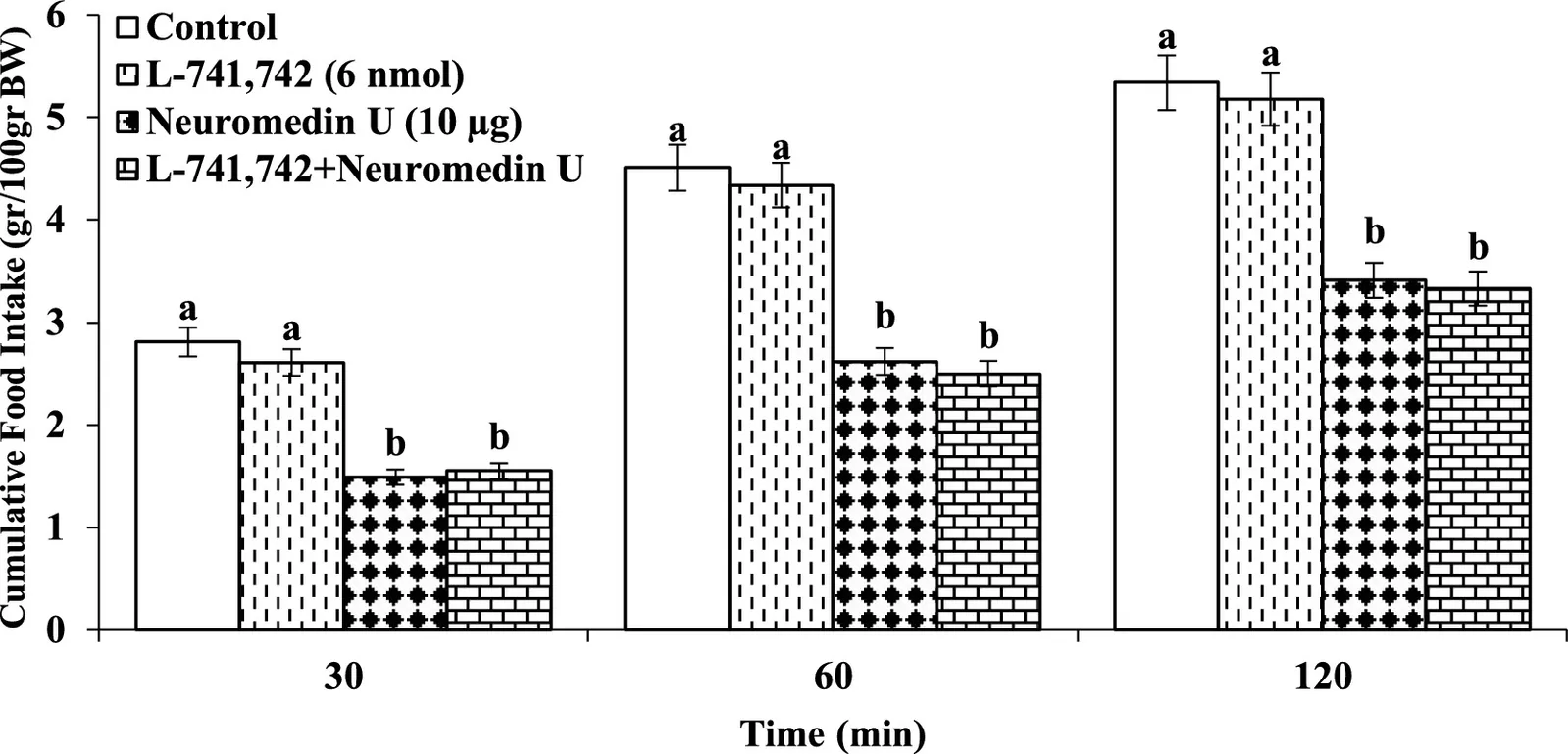
Effect of ICV injection of control solution, l-741,742 (D4 receptor antagonist, 5 nmol), Neuromedin U (10 µg) and a combination of l-741,742 plus Neuromedin U on cumulative food intake (gr/100gr BW) in neonatal broiler chicks (n = 44). Data are expressed as mean±SEM. Different letters (a, b and c) indicate significant differences between treatments at each time (P < 0.05).

### Involvement of muscarinic M1 receptors

Treatment with the M1 receptor antagonist Telenzepine alone did not affect cumulative food intake. However, co-administration of Telenzepine significantly attenuated NMU-induced hypophagia at all evaluated time points (P < 0.05). Food consumption in the Telenzepine + NMU group was significantly higher than in the NMU group but remained lower than in control chicks (Fig. 6).

**Fig. 6:**
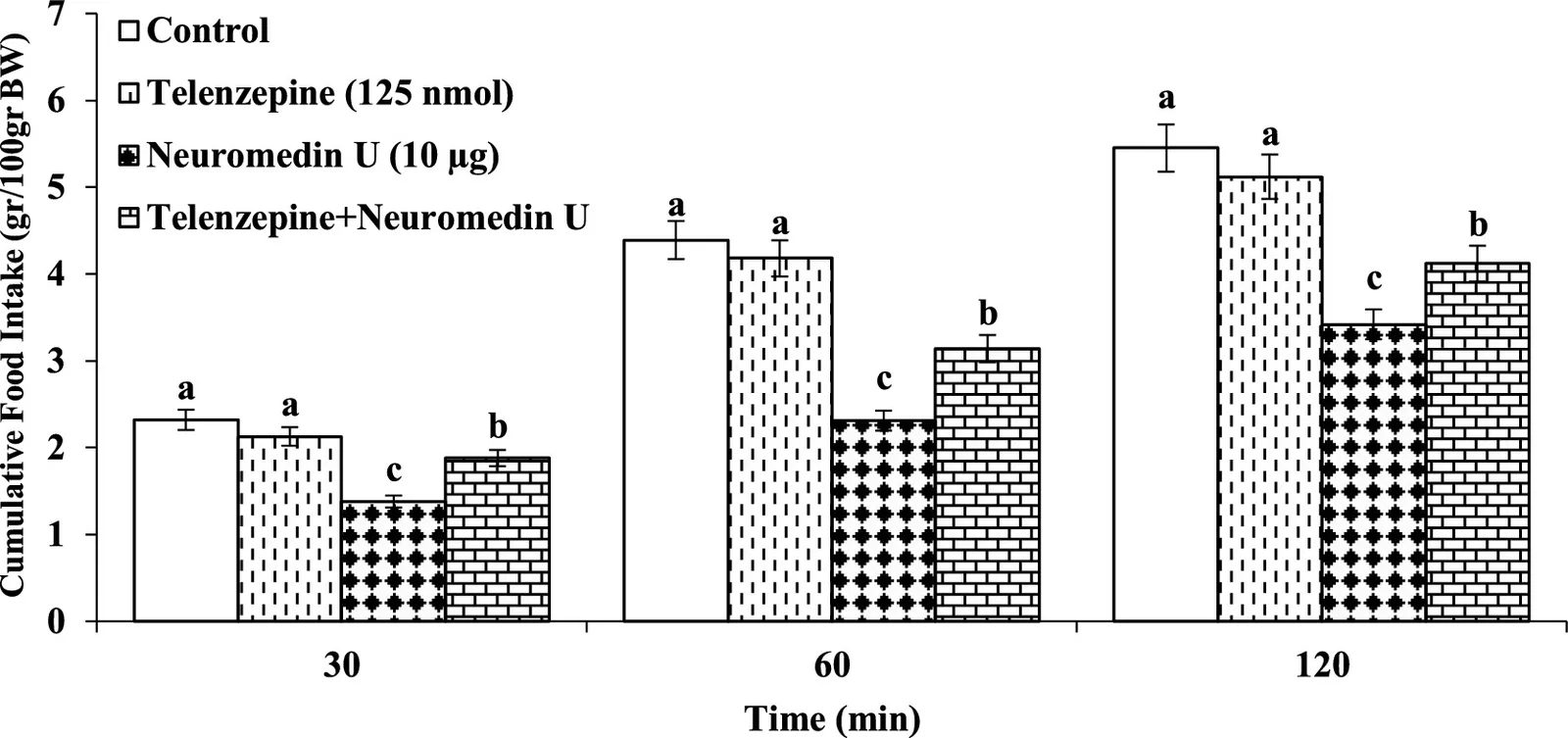
Effect of ICV injection of control solution, Telenzepine (M1 receptor antagonist, 125 nmol), Neuromedin U (10 µg) and a combination of Telenzepine plus Neuromedin U on cumulative food intake (gr/100gr BW) in neonatal broiler chicks (n = 44). Data are expressed as mean±SEM. Different letters (a, b and c) indicate significant differences between treatments at each time (P < 0.05).

### Involvement of muscarinic M2 receptors

Administration of AF-DX116 significantly reduced the anorexigenic effect of NMU. Chicks receiving AF-DX116 before NMU exhibited higher cumulative food intake compared with chicks treated with NMU alone (P < 0.05). These results suggest that M2 receptor signaling contributes to NMU-induced suppression of feeding (Fig. 7).

**Fig. 7:**
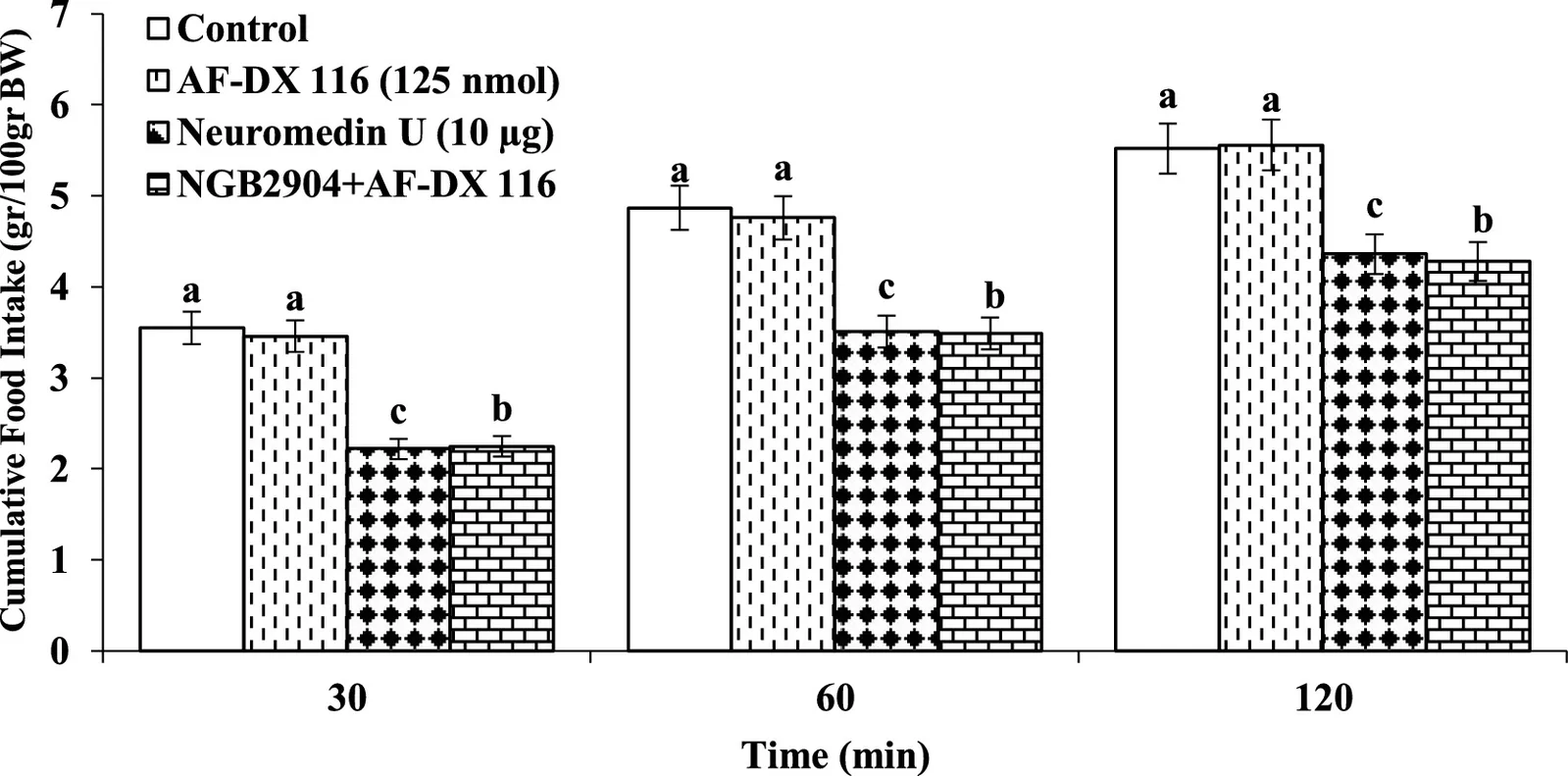
Effect of ICV injection of control solution, AF-DX116 (M2 receptor antagonist, 125 nmol), Neuromedin U (10 µg) and a combination of AF-DX116 plus Neuromedin U on cumulative food intake (gr/100gr BW) in neonatal broiler chicks (n = 44). Data are expressed as mean±SEM. Different letters (a and b) indicate significant differences between treatments at each time (P < 0.05).

### Involvement of muscarinic M3 receptors

Pretreatment with the M3 receptor antagonist 4-DAMP significantly reversed the inhibitory effect of NMU on food intake. Food consumption in the 4-DAMP + NMU group was significantly greater than that observed in the NMU-treated group throughout the experiment (P < 0.05), Suggesting that M3 receptor signaling contributes to the hypophagic response induced by NMU (Fig. 8).

**Fig. 8:**
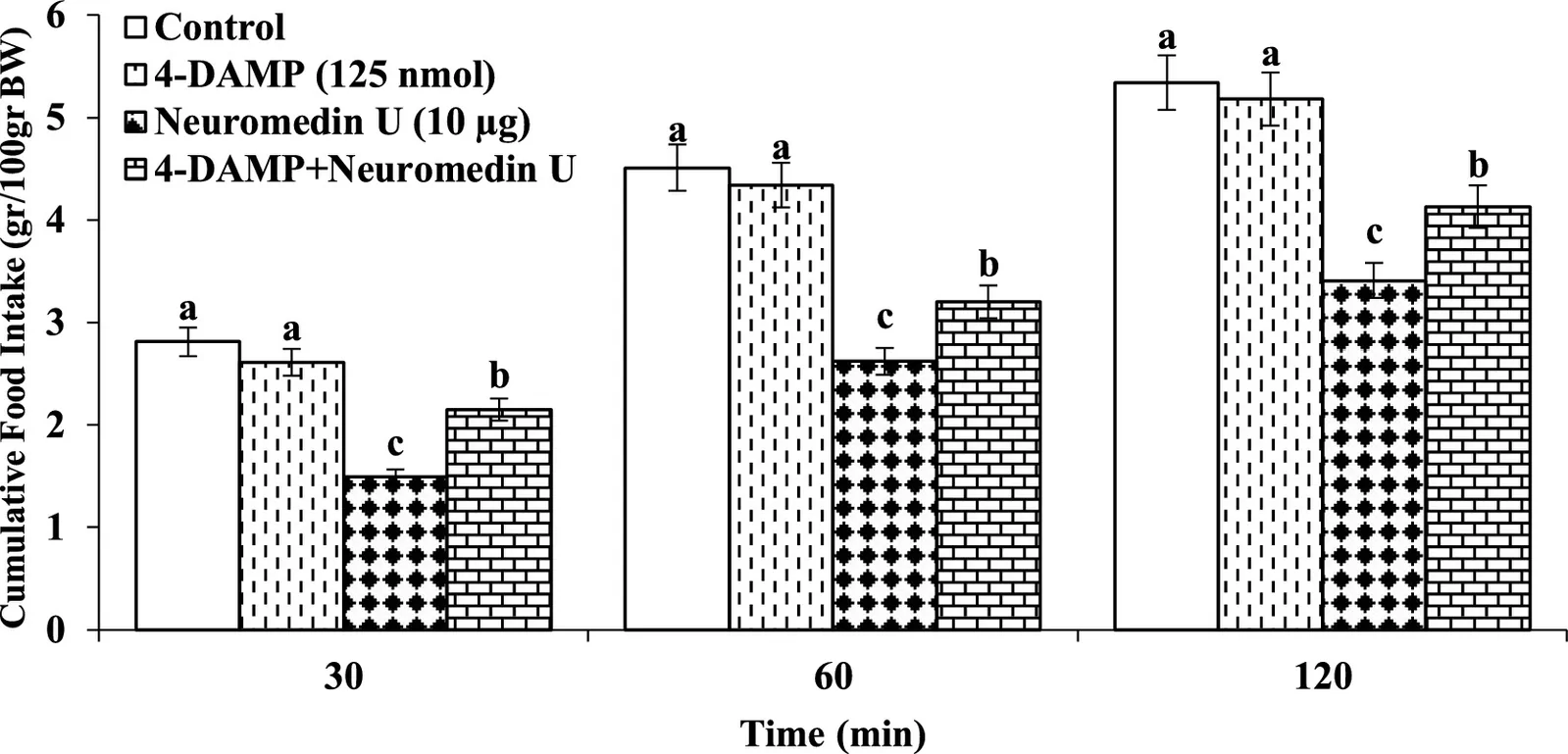
Effect of ICV injection of control solution, 4-DAMP (M3 receptor antagonist, 125 nmol), Neuromedin U (10 µg) and a combination of 4-DAMP plus Neuromedin U on cumulative food intake (gr/100gr BW) in neonatal broiler chicks (n = 44). Data are expressed as mean±SEM. Different letters (a, b and c) indicate significant differences between treatments at each time (P < 0.05).

### Involvement of muscarinic M4 receptors

In contrast, administration of the M4 receptor antagonist PD102807 did not significantly affect the feeding response to NMU. No significant differences were observed between the NMU group and the NMU + PD102807 group at any time point. Therefore, M4 receptors do not appear to participate in the anorexigenic mechanism of NMU in neonatal broiler chicks (Fig. 9).

**Fig. 9:**
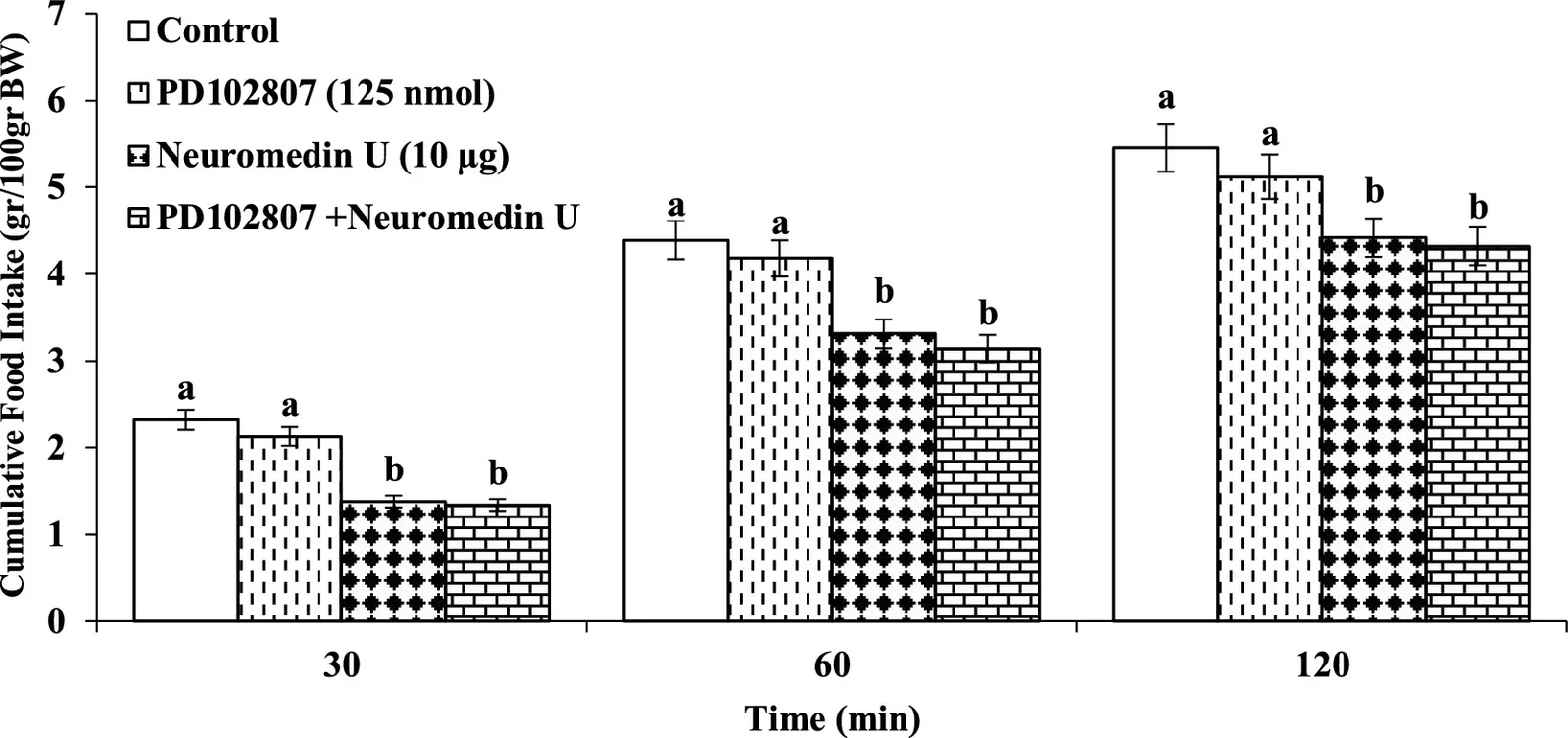
Effect of ICV injection of control solution, PD102807 (M4 receptor antagonist, 125 nmol), Neuromedin U (10 µg) and a combination of PD102807 plus Neuromedin U on cumulative food intake (gr/100gr BW) in neonatal broiler chicks (n = 44). Data are expressed as mean±SEM. Different letters (a and b) indicate significant differences between treatments at each time (P < 0.05).

## Discussion

The present study demonstrates that intracerebroventricular administration of neuromedin U (NMU) suppresses food intake in neonatal broiler chicks in a dose-dependent manner. Pharmacological blockade of dopamine D1 and D2 and muscarinic M1, M2, and M3 receptors attenuated the hypophagic response to NMU, whereas blockade of D3, D4, and M4 receptors did not significantly modify this response. These findings suggest receptor subtype-specific involvement of dopaminergic and muscarinic systems in NMU-induced hypophagia. Importantly, the present pharmacological findings do not establish direct activation of these receptors by NMU or a specific downstream molecular signaling pathway. The observed reduction in food intake following central NMU administration is consistent with previous reports in both mammals and avian species. Earlier studies demonstrated that ICV injection of NMU significantly reduced feeding behavior in chicks, suggesting that the anorexigenic action of NMU is evolutionarily conserved across vertebrates (Kamisoyama et al., 2007). Similarly, NMU has been recognized as a critical neuropeptide involved in energy homeostasis and appetite regulation through activation of NMUR1 and NMUR2 receptors (Hanada et al., 2001). Comparable anorexigenic effects have also been reported in fish species, including Siberian sturgeon, indicating that NMU-mediated feeding suppression is highly conserved among vertebrates (Zhang et al., 2023). The agreement between our findings and previous studies reinforces the physiological importance of NMU as a central regulator of appetite.

A major finding of the current study is the involvement of dopaminergic signaling in NMU-induced hypophagia. Blockade of D1 and D2 receptors significantly attenuated the suppressive effect of NMU on food intake, suggesting that D1 and D2 receptor systems contribute to the behavioral response induced by central NMU administration. Dopaminergic neurotransmission has long been recognized as a critical regulator of feeding behavior, integrating both homeostatic and reward-related mechanisms (Wallace and Fordahl, 2022; Baik, 2021). Activation of D1 and D2 receptors within hypothalamic and mesolimbic circuits influences food-seeking behavior, motivational drive, and energy balance (Wallace and Fordahl, 2022; Volkow et al., 2013). Our results are consistent with the findings of Ghashghayi et al. (Ghashghayi et al., 2024) who demonstrated that dopaminergic pathways contribute significantly to NMU-induced hypophagia in neonatal chicks. However, the present study extends those observations by identifying the specific receptor subtypes responsible for this effect.

Interestingly, blockade of D3 and D4 receptors failed to modify the anorexigenic effects of NMU. The lack of a significant effect of D3 and D4 receptor blockade suggests that these receptor subtypes may not be necessary for the observed hypophagic response under the experimental conditions used in the present study. Previous evidence indicates that D1 and D2 receptors are more directly involved in hypothalamic regulation of appetite, whereas D3 and D4 receptors are more strongly associated with reward processing and motivational behaviors (Wang et al., 2009). Therefore, the lack of involvement of D3 and D4 receptors observed in the present study may reflect functional specialization among dopamine receptor families in avian species. Another important finding was the significant contribution of muscarinic cholinergic receptors to NMU-induced hypophagia. Administration of Telenzepine, AF-DX116, and 4-DAMP significantly attenuated NMU-induced suppression of food intake, indicating that pharmacological blockade of M1, M2, and M3 receptors attenuates the central hypophagic response to NMU. Cholinergic signaling plays a fundamental role in hypothalamic regulation of feeding and energy expenditure through modulation of neuronal excitability and neurotransmitter release (Deem et al., 2022). Among the muscarinic receptor subtypes, M3 receptors have been particularly implicated in metabolic regulation and energy homeostasis. Studies in mammals have demonstrated that disruption of M3 receptor signaling alters feeding behavior, glucose metabolism, and body weight regulation (Tran et al., 2022; Deem et al., 2022). Therefore, the strong attenuation of NMU-induced hypophagia following M3 receptor blockade observed in the current study supports the hypothesis that M3 signaling constitutes an important component of NMU-mediated appetite regulation.

In contrast, blockade of M4 receptors failed to alter NMU-induced feeding suppression. Similar receptor subtype-specific effects have been reported in mammalian studies, where M1 and M3 receptors exert stronger metabolic effects than M4 receptors (Deem et al., 2022). The differential involvement of muscarinic receptor subtypes observed in the present study further emphasizes the complexity of cholinergic regulation of appetite in birds. The mechanisms through which NMU interacts with dopaminergic and cholinergic pathways remain incompletely understood. NMUR2 receptors are highly expressed within hypothalamic nuclei, particularly the paraventricular nucleus (PVN), a major center involved in appetite regulation (Tran et al., 2022). Activation of NMUR2 stimulates intracellular signaling pathways involving phospholipase C, protein kinase C, and calcium-dependent mechanisms, ultimately influencing neuronal activity and neuropeptide release (Tran et al., 2022). Previous studies have shown that NMU suppresses the expression of orexigenic neuropeptides such as neuropeptide Y (NPY) while activating anorexigenic pathways involving corticotropin-releasing factor (CRF) neurons (Cao et al., 2020; Greene et al., 2022). It is therefore plausible that D1/D2 and M1/M2/M3 receptor-mediated signaling acts either downstream of or in parallel with these hypothalamic pathways to amplify the anorexigenic response to NMU.

An additional strength of the present study is the demonstration that multiple neurotransmitter systems contribute to NMU-mediated appetite regulation in poultry. Together with previous evidence implicating serotonergic and GABAergic pathways (Ghashghayi et al., 2024), our findings support the concept that NMU functions as an upstream neuromodulator capable of coordinating several neurochemical networks involved in feeding control. Such integration may be particularly important in rapidly growing broiler chickens, where precise regulation of feed intake is essential for maintaining energy balance and growth performance (Saneyasu, 2024; Morton et al., 2014).

Several limitations should be considered when interpreting the present findings. First, only cumulative food intake was assessed; therefore, the study cannot distinguish specific effects on appetite from nonspecific reductions in feeding associated with stress, malaise, or other behavioral effects. Feeding microstructure, such as meal frequency and meal size, and independent measures of stress or sickness behavior were not evaluated. Second, the observation period was limited to 120 min following acute intracerebroventricular administration. Thus, the present findings should not be extrapolated directly to the physiological role of endogenous NMU during normal feeding or to long-term feed intake and growth performance in commercial broilers. Third, pharmacological antagonist experiments demonstrate receptor-system involvement but cannot establish direct receptor activation by NMU or define the underlying molecular signaling pathways. Finally, pharmacological doses validated in other species may not have identical selectivity or pharmacodynamic properties in the avian brain, representing an additional consideration when interpreting receptor-specific effects.

Overall, the present study provides the first evidence that selective dopaminergic and muscarinic receptor subtypes mediate the anorexigenic effects of NMU in broiler chicks. These findings substantially advance current knowledge regarding the neuroendocrine regulation of feeding behavior in avian species and establish a foundation for future investigations into the molecular and physiological mechanisms underlying appetite control in poultry.

## Conclusion

Central administration of neuromedin U suppresses food intake in neonatal broiler chicks. Pharmacological blockade of dopamine D1 and D2 and muscarinic M1, M2, and M3 receptors attenuated this hypophagic response, whereas blockade of D3, D4, and M4 receptors did not significantly alter feeding suppression. These findings suggest receptor subtype-specific involvement of dopaminergic and muscarinic systems in the acute behavioral response to NMU. However, the present pharmacological approach does not establish direct receptor activation or a specific molecular signaling pathway. Further molecular and physiological studies are required to clarify the mechanisms underlying NMU-mediated regulation of feeding in avian species.

## Consent for publication

Not applicable.

## Availability of data and materials

The datasets generated and/or analyzed during the current study are available from the corresponding author upon reasonable request.

## Funding

This research received no external funding.

## Disclosures

The authors declare that they have no competing interests.

## Author Contributions

Armin Ahmadi: He done data collection during, the rearing period the initial draft of the manuscript and critically reviewed the manuscript for important intellectual content. Shahin Hassanpour: as corresponding author of the paper conceived the research idea, developed the research concept, designed the study protocol and paper framework, planned the overall research methodology, analyzed the data, prepared the figures, prepared and revised the manuscript. Morteza Zendehdel: he contributed to the experimental design, particularly the selection and definition of performance parameters, contributed to the interpretation of the results, and critically revised the manuscript for important intellectual content. Mohaya Farzin: done data collection during, the rearing period the initial draft of the manuscript and critically reviewed the manuscript for important intellectual content. All authors critically reviewed the manuscript for important intellectual content, reviewed and explicitly approved the final version, and agree to be personally accountable for all aspects of the work, ensuring that any questions regarding its accuracy or integrity are appropriately investigated and resolved.

## References

[bib0011] Baik J.H. (2021). Dopaminergic control of the feeding circuit. Endocrinol. Metabolism.

[bib0001] Cao C., Gilbert E.R., Cline M.A. (2020). The anorexigenic effect of beta-melanocyte-stimulating hormone involves corticotrophin-releasing factor and mesotocin in birds. Neurosci. Lett..

[bib0014] Deem J.D., Faber C.L., Morton G.J. (2022). AgRP neurons: regulators of feeding, energy expenditure, and behavior. FEBS. J..

[bib0004] Ghashghayi E., Zendehdel M., Khodadadi M., Rahmani B. (2024). Central dopaminergic, serotoninergic, as well as GABAergic systems mediate NMU-induced hypophagia in newborn chicken. Int. J. Neurosci..

[bib0008] Greene E., Zampiga M., Sirri F., Ohkubo T., Dridi S. (2020). Orexin system is expressed in avian liver and regulates hepatic lipogenesis via ERK1/2 activation. Sci. Rep..

[bib0003] Greene E.S., Abdelli N., Dridi J.S., Dridi S. (2022). Avian neuropeptide Y: beyond feed intake regulation. Vet. Sci..

[bib0017] Hanada R., Nakazato M., Murakami N., Sakihara S., Yoshimatsu H., Toshinai K. (2001). A role for neuromedin U in stress response. Biochem. Biophys. Res. Commun..

[bib0015] Jonaidi H., Abbassi L., Yaghoobi M., Kaiya H., Denbow D., Kamali Y. (2012). The role of GABAergic system on the inhibitory effect of ghrelin on food intake in neonatal chicks. Neurosci. Lett..

[bib0007] Kaiya H. (2024). Update on feeding regulation by ghrelin in birds: focused on brain network. Zoolog. Sci..

[bib0016] Kamisoyama H., Honda K., Saneyasu T., Sugahara K., Hasegawa S. (2007). Central administration of neuromedin U suppresses food intake in chicks. Neurosci. Lett..

[bib0009] Mahdavi K., Zendehdel M., Zarei H., Neuropeptide Y. (2024). An undisputed central simulator of avian appetite. J. Poultry Sci. Avian Diseases.

[bib0020] Morton G.J., Meek T.H., Schwartz M.W. (2014). Neurobiology of food intake in health and disease. Nat. Rev. Neurosci..

[bib0012] Onimus O., Peters K.Z., Naneix F., Gangarossa G. (2026). Dopamine ensembles regulating appetite, feeding, and energy homeostasis. Trends Endocrinol. Metabolism.

[bib0006] Saneyasu T. (2024). Recent research on mechanisms of feeding regulation in chicks. J. Poult. Sci..

[bib0002] Teranishi H., Hanada R. (2021). Neuromedin U, a key molecule in metabolic disorders. Int. J. Mol. Sci..

[bib0013] Tran L.T., Park S., Kim S.K., Lee J.S., Kim K.W., Kwon O. (2022). Hypothalamic control of energy expenditure and thermogenesis. Exp. Mol. Med..

[bib0018] Volkow N.D., Wang G.J., Tomasi D., Baler R.D. (2013). The addictive dimensionality of obesity. Biol. Psychiatry.

[bib0010] Wallace C.W., Fordahl S.C. (2022). Obesity and dietary fat influence dopamine neurotransmission: exploring the convergence of metabolic state, physiological stress, and inflammation on dopaminergic control of food intake. Nutr. Res. Rev..

[bib0019] Wang G.J., Volkow N.D., Thanos P.K., Fowler J.S. (2009). Imaging of brain dopamine pathways: implications for understanding obesity. J. Addict. Med..

[bib0005] Zhang X., Chen S., Tang N., Wang S., Liu Y., Li Y. (2023). Neuromedin U regulates food intake of Siberian sturgeon through the modulation of central and peripheral appetite factors. Br. J. Nutr..

